# Noradrenergic activity as a key target in modulating consciousness

**DOI:** 10.1038/s41598-026-41819-2

**Published:** 2026-03-10

**Authors:** Olympia Karampela, Aurelie Fontan, Lenita Lindgren, Tiziana Pedale, Camilla Brorsson, Fredrik Bergström, Johan Eriksson

**Affiliations:** 1 Umeå Center for Functional Brain Imaging, Umeå University, Umeå, Sweden; 2 Department of Psychology, Umeå University, Umeå, Sweden; 3 Department of Medical and Translational Biology, Umeå University, Umeå, Sweden; 4 Department of Nursing, Umeå University, Umeå, Sweden; 5 Department of Anaesthesia and Intensive Care, Department of Diagnostics and Intervention Sciences, Umeå University, Umeå, Sweden; 6 CINEICC, Faculty of Psychology and Educational Sciences, University of Coimbra, Coimbra, Portugal

**Keywords:** Brain activity, Consciousness, Sedation, Sleep deprivation, Unconscious, fMRI, Leftward bias, Neuroscience, Physiology

## Abstract

How the brain generates conscious experiences remains profoundly mysterious. Pharmacological interventions that alter the state of consciousness have been proposed as a tool to investigate the neural mechanisms of consciousness. However, we have recently demonstrated that the sedative Propofol influences both conscious and unconscious neural processing. Altered arousal, and other pharmacological effects, therefore cannot be assumed a priori to provide information specifically on conscious neural processes. Instead, effects on both conscious and unconscious processes need to be considered. Here we investigated the role of noradrenergic activity in conscious and unconscious visuospatial processing. In Study 1 we used Dexmedetomidine, a sedative that specifically targets α2A noradrenergic receptors. In Study 2, we used sleep deprivation as a natural state of altered arousal, which exerts partially overlapping effects on noradrenaline levels. Unlike Propofol, both Dexmedetomidine and sleep deprivation selectively altered brain activity (fMRI BOLD signal change) during conscious processing. However, the two methods produced distinct effects on visuospatial bias during low arousal: while Dexmedetomidine reduced leftward bias, sleep deprivation increased leftward bias. These differential effects on spatial bias were explained by an unexpected increase in sympathetic drive, as indexed by increased activity in the central autonomic network and in heart rate, from sleep deprivation, that indicate increased rather than decreased noradrenaline levels during task performance. Together, these findings emphasize noradrenergic activity as a target for pharmacological manipulations of consciousness, which could open a window to its neurophysiological underpinnings.

**Supplementary Information:**

The online version contains supplementary material available at 10.1038/s41598-026-41819-2.

## Introduction

Despite extensive research, the mystery of how the brain generates conscious experiences persists. Central to this challenge is the multifaceted nature of consciousness, which manifests in at least two key dimensions: the content and the level of consciousness. Content represents the subjective, phenomenal quality associated with experiences, such as the “feeling of pain” or “the experience of redness”^1^. The level of consciousness refers to arousal or wakefulness, exemplified by states such as coma, sleep, or anaesthesia^2–4^. These two aspects have primarily been examined in isolation, sparking considerable debates on how to conceptualize their relation^5–10^.

In pursuit of a mechanistic understanding of consciousness, pharmacological interventions have been proposed. Typically, these interventions involve manipulating the level of consciousness through various pharmacological agents to identify differences between conscious and unconscious states, or between high and low levels of arousal, that generalize across drugs^11–13^. However, we have recently demonstrated that the sedative Propofol altered both conscious and unconscious neural processing^14^. This finding challenges the common assumption that sedatives selectively alter (content) consciousness.

While Propofol apparently alters both conscious and unconscious stimulus processing, other sedatives may have distinct, more targeted effects. For example, previous research has shown a relationship between noradrenaline levels and conscious perception. Specifically, Gelbard-Sagiv and colleagues found that altered levels of noradrenaline changed sensitivity and accuracy of visual perception and related EEG and fMRI signals during threshold detection and discrimination tasks^15^. However, since they did not report signal change for unconscious stimuli, it remains unclear if the effects were specifically related to conscious perception or if the altered noradrenaline levels also affected unconscious stimulus processing.

Here we aim to further clarify the relation between “level” and “content”, and specifically the role of noradrenaline on conscious experience. In Study 1, we measured the effects of Dexmedetomidine (Dexdor), a selective α2A adrenergic receptor agonist, on fMRI blood-oxygen-level dependent (BOLD) signal associated with conscious and unconscious processing of visuospatial stimuli. However, exploring consciousness through anaesthesia is challenging. Anaesthetic drugs may have multiple effects on the brain which complicates the interpretation of outcomes. To address this issue, we in Study 2 used sleep deprivation to compare its effects to those induced by Dexdor. Interestingly, Dexdor has been reported to activate sleep-promoting pathways by decreasing the firing of noradrenergic locus coeruleus neurons in the brain stem. Qualitatively, Dexdor induces a sedative response that exhibits properties which more closely resemble natural sleep compared with other sedatives^16^. Considering the similarity between Dexdor’s sedative mechanism to that of natural sleep, we anticipated similar effects from Dexdor sedation and sleep deprivation.

## Results

**Fig. 1:**
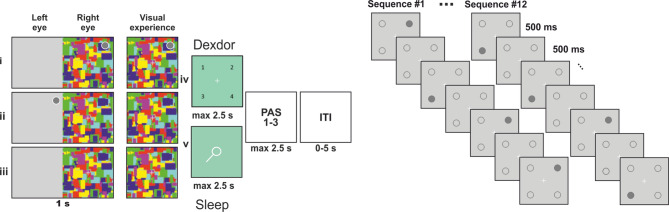
The visuospatial task was composed of three presentation conditions. In each condition, Mondrians were displayed to the dominant (right) eye. (i) In the conscious condition, a grey disc was presented to the right eye, superimposed on the Mondrians. (ii) In the suppressed condition, the disc was presented to the left eye, superimposed on a grey background, and (iii) in the absent condition only the grey background without the grey disc was presented to the left eye. The central column illustrates the visual experience of participants. After the 1 s Mondrian + disc presentation (note that the disc was presented for 500 ms during supressed trials, see Methods), a probe appeared prompting participants to use a keyboard to choose the button corresponding to the relevant quadrant (Dexdor study). For the Sleep study a probe pointing to one of the four quadrants was presented for a maximum of 2.5 s or until participants decided whether it was pointing to the correct location of the disc (yes/no response). The probe could point to the correct location of the sample (match), or to an incorrect location (non-match). Participants then estimated, within 2.5 s, their conscious experience of the disc on a three-point perceptual awareness scale. Finally, trials were separated by an inter-trial interval (ITI). Note that for the purpose of illustration only, the grey dot is encircled in white. Note that the difference in response mode between studies leads to different performance measures, such that hits – misses and hits – false alarms were analysed for study 1 and 2, respectively. The metronome task was performed before the visuospatial task and was used to measure the effect of the change behaviorally and to generate fMRI data on visuospatial stimulation independently from the visuospatial task. To this end, a gray disc was presented in a semi-blocked fashion in a specific quadrant throughout a sequence of 30 metronome “clicks” for the sleep study and 20 metronome “clicks” for the Dexdor study.

The participants performed a simple visuospatial task consisting of noting the location of a grey disc presented in one of the quadrants of a display. Visual perception of the disc was manipulated with continuous flash suppression (CFS) (Fig. 1). CFS refers to an interocular suppression effect wherein a static visual stimulus (the target) presented to the non-dominant eye is suppressed from awareness because of a dynamically changing high-contrast pattern presented to the dominant eye^17^. On each trial participants also reported their conscious experience of the disc on a three-point perceptual awareness scale (PAS), from 1: no visual experience, to 3: clear or almost clear visual experience of the disc. As a reference condition we also included “absent” trials where no target stimulus was presented. This enabled us to isolate the BOLD signal change specifically related to visuospatial neural processing of conscious and suppressed stimuli and to exclude the general, non-specific effects from the manipulations of arousal, including e.g., basal physiological processes. A visual metronome task was performed in separate blocks from the visuospatial task. The metronome task was used to define regions of interest for analysis of the visuospatial task, and as a behavioural measure of participants’ state of arousal by quantifying timing performance^18,19^ (Fig. 1).

In Study 1 arousal was manipulated by administering two levels of the sedative Dexdor: a low sedation level (LS; 0.01 µg/kg/h), and a moderate sedation level (MS), which was individually adjusted ranging from 0.6 to 0.8 µg/kg/h (mean ± SD: 0.68 ± 0.07 µg/kg/h). Importantly, the moderate Dexdor level was adjusted to make participants drowsy but remain awake and able to perform the tasks – crucial to allow a characterization of effects on both conscious and suppressed stimuli. In Study 2, manipulation of arousal involved depriving participants of a full night’s sleep (See SI Methods for a detailed description of the procedure for both studies). Changes in PAS during low arousal levels are detailed in the SI Results. In further analyses, only trials with PAS = 1 (suppressed or absent) and PAS = 3 (conscious) were included.

Both Dexdor sedation and sleep deprivation led to increased response-timing variability during the metronome task (Dexdor LS: *M* = 109 ms, *SD* = 30; MS: *M* = 120 ms, *SD* = 27; *t*_24_ = 3.31, *p* = 0.03; non-sleep deprived: *M* = 93 ms, *SD =* 25; sleep deprived: *M* = 114 ms, *SD* = 30; *t*_30_ =3.70, *p* = 0.0009). A 2 × 2 mixed-effects ANOVA demonstrated a significant main effect of arousal (F_1, 54_ = 20.52, *p* = 0.000033), with no significant interaction between arousal and study (F_1, 54_ = 2.30, *p* = 0.135). Thus, the pattern of increased timing variability was comparable across studies, suggesting a similar reduction in arousal.

Nevertheless, participants consistently performed the visuospatial task with high accuracy regardless of arousal level for conscious trials in Study 1 (hits – misses; mean ± SD: LS = 1.00 ± 0.02; MS = 1.00 ± 0.01) and Study 2 (hits – false alarms; mean ± SD: non-sleep deprived = 0.96 ± 0.03; sleep-deprived = 0.96 ± 0.04). Performance on suppressed trials was also not affected by arousal levels and was significantly better than chance in Study 1 (mean ± SD: LS = 0.06 ± 0.08, *t*(24) = 3.46; *p* = 0.002; MS = 0.05 ± 0.05, *t*(24) = 4.80, *p* = 0.000069), and at chance level in Study 2 (mean ± SD: non-sleep deprived = 0.04 ± 0.11, *t*(30) = 1.90; *p* = 0.07; sleep deprived = 0.01 ± 0.10, *t*(30) = 0.75, *p* = 0.46).

### Brain activity during low-sedation and non-sleep-deprived, i.e., high arousal, trials

**Fig. 2:**
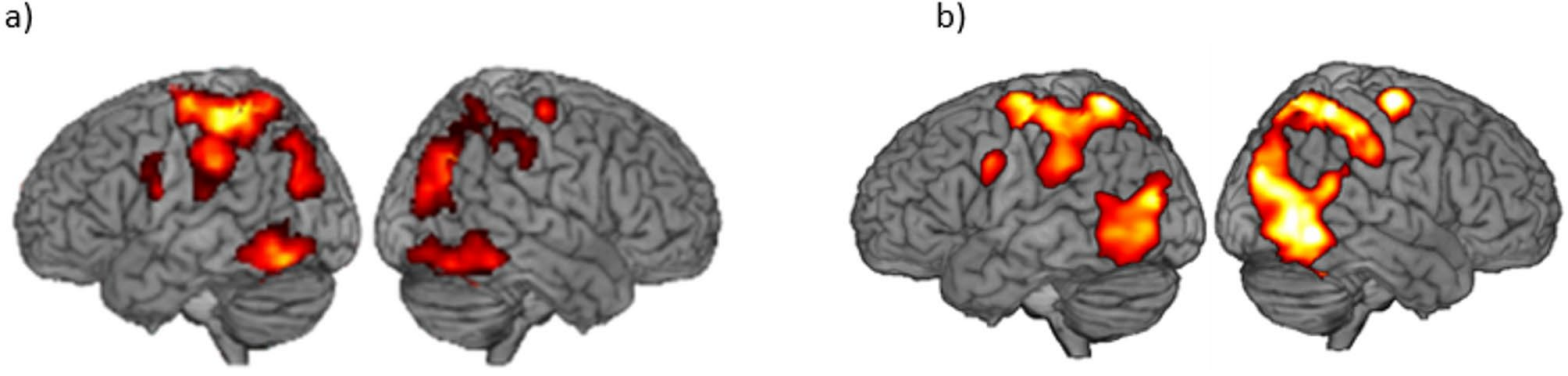
(**a**) BOLD signal change for Conscious > Absent for the Dexdor study under high arousal (**b**) BOLD signal change for Conscious > Absent for the sleep deprivation study under high arousal.

Following our previous analysis pipeline^14^, we first investigated the neural response related to visuospatial processing at a high level of arousal (HA) to verify task-related BOLD signal change in brain areas consistent with visuospatial processing for both conscious and suppressed stimuli, which could potentially be modulated by arousal. Whole-brain univariate analyses of fMRI data, contrasting the conscious HA to the absent HA condition, revealed significant BOLD signal change in the expected regions related with visuospatial processing, including superior parietal cortex, intraparietal sulcus, and occipitotemporal regions bilaterally for both studies (*p* < 0.05 FWE, cluster-defining threshold = 0.001) (Fig. 2). Given the issue of multiple comparisons associated with whole-brain analyses, combined with the expectedly weak signal change during processing of stimuli suppressed with continuous flash suppression, we did not expect to find any significant BOLD signal change at the whole-brain level for suppressed > absent trials. We nevertheless performed the analysis to verify that we would not miss potentially relevant signal change when moving forward with region-of-interest based analyses. As expected, nothing was significant in the whole-brain analysis when comparing suppressed HA > absent HA (*p* < 0.05 FWE, cluster-defining threshold = 0.001). We then used data from the metronome task to generate regions of interest (ROIs) independent of the visuospatial task, in which analyses of BOLD signal change during the visuospatial task were performed. Considering the retinotopic organization of visual cortex, we compared left- > right-sided stimuli to define a region in the right hemisphere, and right- > left-sided stimuli for a region in the left hemisphere. To generate ROIs that approximate the ROI sizes in our previous study^14^ we here used an ad-hoc threshold of *p* < 0.0001 uncorrected. This resulted in a right-hemisphere region located in lateral occipitotemporal cortex (MNI coordinates Dexdor: 44–70 -8; sleep: 48–76 -2) and a corresponding left-hemisphere region (Dexdor: -44 -76 -4; sleep: -44 -72 2), that overlapped with the regions reported previously.

For the visuospatial task, a significant Hemisphere-by-Stimulus Side interaction confirmed a stimulus-related signal change for conscious and suppressed trials in both Study 1 (conscious: F_1_, _24_ = 84.62, *p* = 2.4 × 10^− 9^, suppressed: F_1_, _24_ = 6.00, *p* = 0.022) and Study 2 (conscious: F_1_, _30_ = 32.25, *p* = 0.000003, suppressed: F_1_, _30_ = 9.86, *p* = 0.004), which could be modulated by sedation/sleep deprivation.

### Effects from sedation and sleep deprivation on brain activity

There is extensive evidence to suggest that the right hemisphere is dominant for spatial attention at the population level, resulting in left-hemifield neglect in stroke patients with lesions in the right hemisphere^20,21^ and a leftward bias (a.k.a. pseudoneglect) in healthy individuals^22–24^. Moreover, a growing body of research associates low levels of arousal with a spatial shift of attention towards the right side (see ref.^25^for meta-analysis). Therefore, changes in arousal are expected to cause shifts in the spatial bias of attention, consistent with our previous findings using Propofol sedation and an identical visuospatial task as used here^14^. We therefore calculated a measure of spatial bias from the BOLD signal change in the left and right ROIs elicited by stimuli appearing in the left/right visual field, separately for conscious and suppressed stimuli. Bias was defined as the average signal change across left and right ROIs from left-sided vs. right-sided stimuli (i.e., a main effect of stimulus side if considering stimulus side and hemisphere as two factors in a 2 × 2 ANOVA). The key question here is whether the spatial bias will change for both conscious and suppressed stimuli, as it did during Propofol sedation, or if it will change only for conscious stimuli, as would be expected if noradrenaline specifically alters conscious neural processing.

**Fig. 3:**
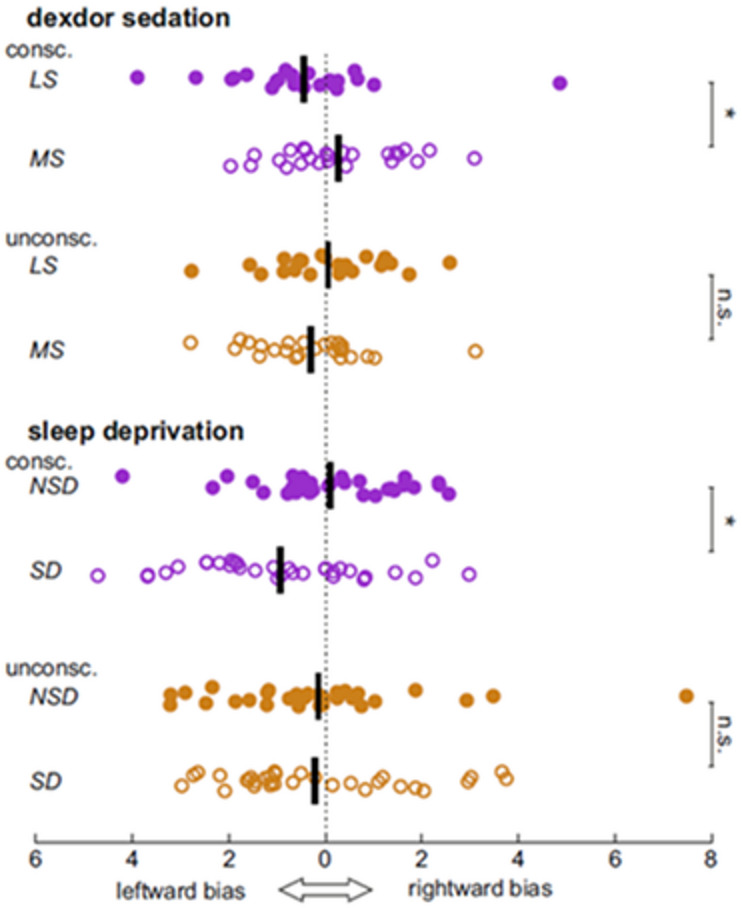
Statistically significant changes in spatial bias during conscious stimuli resulted from both moderate Dexdor sedation and sleep deprivation. While Dexdor sedation led to reduced leftward bias, sleep deprivation led to increased leftward bias. Importantly, neural processing of suppressed stimuli was unaffected by both Dexdor sedation and sleep deprivation. LS = low sedation, MS=moderate sedation, NSD = non-sleep deprived, SD=sleep deprived.

Both Dexdor sedation and sleep deprivation led to changes in the spatial bias for conscious stimuli (Fig. 3; Dexdor: F_1, 24 =_ 5.25, *p* = 0.031, Sleep: F_1, 30_ = 5.31, *p* = 0.028). Importantly, no significant effects were seen for suppressed stimuli during Dexdor sedation (F_1, 24_ = 1.30, *p* = 0.27) or sleep deprivation (F_1, 30_ = 0.08, *p* = 0.78).

However, the nature of the altered spatial bias during conscious trials differed between the two studies. Specifically, moderate Dexdor sedation led to a decreased leftward bias compared to low sedation, in line with previous research^26^. By contrast, sleep deprivation led to increased leftward bias. This reversed pattern for sleep deprivation was unexpected, especially given the link between the pharmacodynamics of Dexdor and natural sleep mechanisms suggested by previous research^16^.

To explore this, we hypothesised that participants may intentionally try to uphold sustained attention during the task and thereby counteract the lethargic effects from the interventions. Such “try-to-stay-alert” effort is expected to involve activity increase in the dorsomedial prefrontal cortex (dmPFC), which plays a central role in modulating cognitive control in response to increasing demands^27^, together with other nodes of the central autonomic network (CAN)^28^. This is in turn expected to cause release of noradrenaline via the sympathetic branch of the autonomic nervous system^29^, and as a consequence lead to increased heart rate. An effort to stay alert may have the desired effect in the context of sleep deprivation but not if noradrenaline release is reduced pharmacologically, as in the participants under Dexdor sedation.

**Fig. 4:**
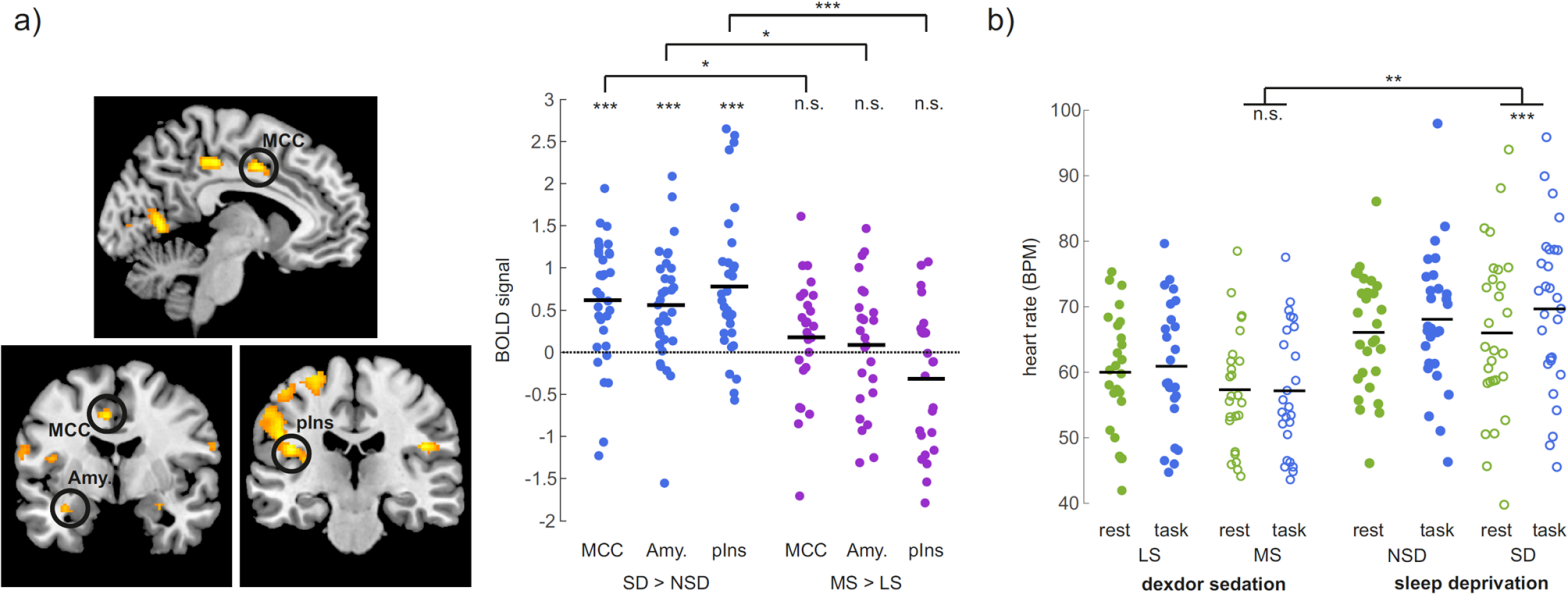
(**a**) Increased BOLD signal in the posterior insula (pIns; MNI xyz = -38 -12 12; *p* < 0.05 FWE, CDT = 0.001), in midcingulate cortex (MCC: -8 -2 42) and left amygdala (Amy; -34 -4 -18) (*p* < 0.001 uncorrected), after sleep deprivation. (**b**) Significantly increased heart rate was observed for the sleep-deprived participants during the visuospatial task compared to resting state, in contrast to the participants in the Dexdor study. The elevated heart rate indicates increased sympathetic tone and a concurrent increase in noradrenaline release. Counterintuitively, this indicates that noradrenaline levels increased rather than decreased when performing a task after sleep deprivation.

Consistent with our predictions, we observed significantly greater CAN activity in the sleep-deprived condition compared to the non-sleep-deprived condition, whereas no such pattern was found in participants under Dexdor sedation (Fig. 4). Specifically, averaged across all trial types (conscious, suppressed, and absent) there was increased BOLD signal in the posterior insula (MNI xyz = -38 -12 12; *p* < 0.05 FWE, CDT = 0.001), in midcingulate cortex (MCC) (-8 -2 42) and left amygdala (-34 -4 -18) at a more liberal threshold (*p* < 0.001 uncorrected), after sleep deprivation (Fig. 4a). Significant differences in BOLD activation were found between the sleep deprivation and moderate sedation in all three regions (posterior insula: *t* (53) = 4.52, *p* = 0.000012; MCC: *t* (53) = 2.30, *p* = 0.027; amygdala: *t* (53) = 2.40, *p* = 0.017).

Increased sympathetic activation was also reflected in an increased heart rate observed during the visuospatial task compared to rest in the sleep-deprived participants (Task: *M* = 69.6, *SD* = 12.5; Rest: *M* = 65.9, *SD* = 12.7; *t* (28) = 4.19, *p* = 0.00026). In contrast, moderately sedated participants showed no significant difference in heart rate between task and rest (Task: *M* = 56.9, *SD* = 9.6; Rest: *M* = 57.0, *SD* = 8.9; t *(*24) = − 0.23, *p* = 0.82). This was further confirmed by a mixed-effects ANOVA which revealed a significant interaction between study (sleep vs. dexdor) and task (task vs. resting), *F* (1, 51) = 11.82, *p* = 0.0018. (Fig. 4b).

To verify the specificity of the observed effect from arousal and the difference across studies, we conducted a 2 × 2 × 2 mixed-effects ANOVA including study, arousal, and consciousness as factors. This analysis revealed a significant study x arousal interaction (F_1,__54_ = 4.33, *p* = 0.042) demonstrating that the effect from arousal on spatial bias differed between the two studies. Moreover, the study x arousal x consciousness interaction was significant (F_1, 54_ = 4.40, *p* = 0.041), demonstrating that this study-dependent difference also differed significantly as a function of consciousness.

To explore if low arousal might alter brain activity related to conscious neural processing outside the current ROIs, we also performed a whole-brain analysis of [conscious HA – absent HA] > [conscious LA – absent LA] (*p* < 0.05 FWE, cluster-defining threshold = 0.001). There was no significant signal change for either study. The corresponding analyses for suppressed trials did not reveal any significant signal change.

## Discussion

Here, we have shown that the effects of Dexdor sedation and sleep deprivation on brain activity were seen exclusively for conscious neural processing. These results align with prior findings by Gelbard-Sagiv and colleagues, where both increased and decreased noradrenaline (NA) levels altered conscious stimulus processing^15^. Importantly, by measuring effects on both conscious and suppressed stimuli we here provide the first pharmacological neuroimaging evidence for the involvement of NA specifically in conscious visuospatial processing – a specificity that replicated across two separate experiments.

Interestingly, certain cellular mechanisms described in previous research appear to play a crucial role in brain networks that could support conscious perception, with noradrenaline acting as a key modulator. For example, when the locus coeruleus is phasically active, certain stimuli are more likely to reach consciousness by triggering NA “hot spots”^30^. At these local hot spots, NA release and glutamate work together, amplifying the neural representation of prioritized stimuli while weaker or less relevant signals elsewhere are suppressed by NA through lateral and auto-inhibitory processes. By regulating the signal-to-noise ratio, NA could help make prioritized stimuli more distinct, increasing their chances of reaching conscious awareness.

Another possible mechanism involving noradrenaline in conscious experience is apical amplification in pyramidal neurons, where NA could enhance sensory integration at the apical dendrites, selectively amplifying relevant inputs and increasing their likelihood of reaching conscious awareness. The extent of apical amplification is modulated by the level of arousal. In the awake state, increased arousal leads to higher amplification allowing relevant stimuli to become conscious, while other stimuli remain unconscious unless further amplified^31^. Importantly, while these mechanisms suggest how NA could modulate arousal to shape conscious experience, they do not imply that NA is the sole factor involved or that a specific level of adrenergic arousal is sufficient for conscious perception. Rather, they suggest that NA could prepare the brain to amplify specific contents to enter conscious awareness.

Notably, the characterization of NA as a gain amplifier^32^ also links our findings to “ignition” theories of consciousness, such that NA may be part of the neural underpinnings of conscious ignition^33^. However, how, and whether, noradrenaline interacts with other neuromodulators to enhance conscious experience remains unclear. Moreover, the mounting evidence for unconscious attention^34^ raises an important question of whether the mechanisms described above are related to attention or consciousness, or their interaction.

Developing brain asymmetry has been shown to consistently influence several cognitive outcomes^35^, and considerable evidence supports an attentional bias towards the left both involving healthy individuals (pseudoneglect^22,24^) and neurological patients (hemispatial neglect^20,21^). This observed asymmetry in visuospatial attention has long been attributed to the specialization of the right hemisphere in mediating spatial attention^36^. Furthermore, a growing body of research links decreased arousal levels with a spatial shift of attention towards the right side^25^.

Consistent with this, moderate Dexdor sedation significantly diminished the typical leftward bias. However, sleep deprived participants showed an increased leftward bias. While both Dexdor sedation and sleep deprivation were associated with increased response variability in the metronome task, only sleep deprivation was linked to significantly greater activation in the dmPFC and other nodes of the central autonomic network, as well as elevated heart rate during task execution, suggesting the engagement of compensatory arousal mechanisms. An interaction between arousal and attentional bias can be understood within the VAN/DAN framework, which distinguishes between the bilateral dorsal attention network (DAN) and the right-lateralised ventral attention network (VAN)^20^. Connectivity between VAN/DAN has been linked to leftward bias^37^. Arousal has been shown to be predominantly right lateralised^38,39^. Given this right-hemisphere dominance for both VAN/DAN interactions and arousal, increases in arousal are expected to strengthen a relative leftward shift in spatial attention.

These findings indicate that sleep-deprived participants made active efforts to maintain wakefulness potentially triggered by external cues such as prompts from the experimenter to stay alert, internal motivation to complete the task, or even stress induced by task demands. Circadian influences on altered interocular suppression, changes in eye dominance, or oculomotor control, following sleep loss may also have contributed to changes in spatial bias, although the specificity to conscious stimuli suggests otherwise. While we cannot address these factors in the present study, future work should aim to disentangle them to clarify their specific effects on spatial bias in relation to altered arousal.

An important consideration when interpreting results based on subjective awareness measures is that the results can be confounded by overestimation of unconscious processing due to a possibly conservative response bias. In this study, we chose the Perceptual Awareness Scale (PAS) for its ability to provide a measure of consciousness at a trial level that is not captured by objective measures. Objective measures have low face validity and risk underestimating unconscious effects by misclassifying unconscious processing as conscious^40^. Although subjective measures are widely endorsed^41^ and well-motivated in the research of consciousness^42^ their inherent limitations must be considered when interpreting the present findings. Notably, suppressed stimuli in the sleep study were unconscious according to both subjective and objective criteria.

Interestingly, our current findings contrast with those of Fontan et al. ^14^, where we reported that low arousal from Propofol influenced both conscious and unconscious stimulus processing. This discrepancy suggests that Propofol may have drug-specific effects on unconscious processes, consistent with the notion that sedative-induced modulation of brain activity is not uniform across different agents^43^. While we previously interpreted the Propofol findings as demonstrating a dissociation between the level and content of consciousness, the similar effect on conscious trials observed across the three manipulations of arousal (i.e., Propofol, Dexdor, and sleep deprivation) aligns with theories of consciousness that emphasize the integration of both the level (arousal) and content (subjective experience) of consciousness^5,44^. Whether Propofol is the odd one out by affecting both conscious and unconscious stimulus processing, which suggests a possible association between level and content of consciousness after all, or if the effects of noradrenaline is indeed as selective as the current findings suggest, which suggests that noradrenaline and not arousal level in general alters (content) conscious stimulus processing, should be a prioritized topic for further research. In either case, further research at multiple levels (system, network, cellular) on how noradrenaline alters conscious stimulus processing promises to further our understanding on the neural correlates of consciousness.

## Materials and methods

A total of 42 and 44 healthy, right-handed adults, respectively for the Dexdor and Sleep study, were recruited from the Umeå University campus via posters and internet advertisements. All participants had normal or corrected-to-normal vision, right-eye dominance, provided written informed consent, and received financial compensation (2000 SEK) for their participation. Both studies employed a within-subjects design. In the Dexdor study, one participant was excluded due to high blood pressure, and three did not complete both MRI sessions. Five participants received a modified training sequence that led to aberrant performance during scanning and were therefore excluded. Additionally, eight participants were removed from data analysis due to either excessive head movement during fMRI (*n* = 1) or failure to follow task instructions (*n* = 7), resulting in a final sample of 25 individuals (mean age ± SD: 28.2 ± 5.9 years; 10 males). In the Sleep deprivation study, 13 participants were excluded from data analysis, either due to excessive head movement during fMRI scanning (*n* = 4) or failure to follow task instructions (*n* = 9), yielding a final sample of 31 individuals (mean age ± SD: 27.2 ± 4.7 years; 12 males; see SI Methods Fig. 1 for exclusion flowchart). Both studies received approval from the Swedish Ethical Review Authority (reference number 2019 − 01613). All experiments were performed in accordance with relevant guidelines and regulations.

### Paradigm and stimuli

For the Dexdor study, during fMRI scanning, participants performed a visuospatial task, under continuous flash suppression (CFS), composed of 160 trials equally distributed in two blocks and divided into three presentation conditions: 40 conscious, 68 suppressed, and 52 absent trials for each sedation level. Each trial was randomly chosen from one of the three conditions. For the sleep study during each fMRI scanning session, participants performed a slightly modified version of the visuospatial task, under continuous flash suppression (CFS), composed of 180 trials equally distributed in 2 blocks and divided into three presentation conditions: 56 conscious, 66 suppressed, and 58 absent trials. Each trial was randomly chosen from one of the three conditions.

A visual metronome task was also performed and used as a behavioural measure of participants’ state of arousal. Participants had to synchronize finger taps to visual isochronous metronome sequences presented to their dominant eye and were requested not to follow the beat by moving other body parts or using covert counting. The stimulus was the same disc as for the visuospatial task but presented on the grey background with empty dotted circles reflecting the four possible positions of the stimulus.

For CFS, a mirror stereoscope was used to isolate visual input from the left and right side of the screen to participants’ corresponding eyes. For suppressed trials, the target stimulus (grey disc; size = 0.6º) was presented for 500 ms to the non-dominant (left) eye while colored squares of random composition (“Mondrians”; size = 4.2° × 4.2°) were flashed (10 Hz) to the dominant eye to suppress conscious experience of the disc. Mondrians were flashed for 500 ms longer than the disc’s presentation, minimizing the risk of adaptation after-effects. To maximize stimulus intensity during suppressed trials, contrast between the disc and the gray background was increased or decreased every 10 trials depending on how many times participants reported the disc as seen. That way, the proportion of actual subjectively experienced disc presentations was 80%. There were 17 possible contrast values. The difference between each contrast consisted of an increase or a decrease in RGB value of 2 (range = 174–206; background = 210). For conscious trials, the disc (RGB = 198) was superimposed on Mondrians, presented to the dominant eye, and was thus consciously seen. For “absent” trials, used as reference condition, Mondrians were presented to the dominant eye while an empty grey background (4.2° × 4.2°) was presented to the non-dominant eye.

**Dexdor**: For conscious and suppressed trials, the disc was presented in one of the four quadrants of the screen. The position was randomly selected from a pre-specified list where positions were counterbalanced within each condition. After the disc presentation, a probe was presented, and participants had to decide the disc’s location among the four quadrants. Participants replied using a keyboard and were instructed to press the key corresponding to the quadrant as follows: 1 for quadrant up left, 2 up right, 3 down left, and 4 down right. For suppressed trials participants were instructed to guess on the first alternative that came to mind and to avoid always pressing the same key. After the probe, participants estimated their conscious experience of the disc on a three-point perceptual awareness scale (PAS), from 1: no visual experience to 3: clear or almost clear visual experience of the disc. For probe and PAS, participants had to reply within a limit of 2.5 s after which the experiment automatically continued to the next trial. The inter-trial interval (ITI) was adjusted according to participants’ response time in a way that two trials were always separated by 5 s. For the metronome task one trial consisted of a 500 ms stimulus presentation followed by a 500 ms ITI. In total, participants completed eight sequences of 20 trials (maximum of 200 visual presentations) where the stimulus was presented with a 1-Hz tempo. The stimulus’ position within each block was selected in a pseudo-random order in a way that a block mainly (85% of the trials) consisted of stimuli appearing in one quadrant. Participants received feedback about their performance at the end of each sequence.

**Sleep**: In the Sleep study for conscious and suppressed trials, the disc was presented in one of the four quadrants of the screen. After the disc presentation, a probe was presented, pointing either to the same spatial location as the disc (match) or to another spatial location (non-match). Participants had to decide whether the probe was pointing to the disc’s location (yes/no). For suppressed trials and absent trials, participants were instructed to guess on the first alternative that came to mind. There was a 50% chance that the probe pointed to the disc location. After the probe, participants estimated their conscious experience of the disc on the same three-point perceptual awareness scale as in the Dexdor study. The visual metronome task was also performed as it is described for the Dexdor study where participants completed eight sequences of 30 trials.

### Procedure MRI data collection

Dexmedetomidine infusion started right before participants were placed in the scanner bore. Participants began the experiment with either the low (“LS”) or the moderate (“MS”) sedation. In the final sample, 15 participants started with LS and 10 with MS. A certified intensive-care nurse with specific responsibility for pharmacological administration and monitoring was present throughout the session, and complete resuscitation equipment was always available. See SI methods for details regarding individual adjustments of Dexmedetomidine levels.

The session started with structural imaging (T1, T2 FLAIR and T2 PROPELLER sequences) and one task-fMRI sequence during which participants performed the metronome task, so that Dexmedetomidine levels could stabilize before the following fMRI scanning. Then, one resting-state fMRI sequence was run for the use of another study and will not be further reported here, and task fMRI during which participants performed the CFS followed. During task-fMRI, participants performed two 7-min blocks of the visuospatial task. Finally, to quantify potential changes in regional cerebral blood flow (CBF), the MRI session included one pulsed arterial spin-labeling (ASL) sequence. The two MRI sessions were separated by 7–10 days.

MRI data were collected with a General Electric 3 Tesla Discovery MR750 scanner (32-channel receive-only head coil). High-resolution T1-weighted structural image was collected FSPGR with TE = 3.2 ms, TR = 8.2 ms, TI = 450 ms, and flip angle = 12°. Task-fMRI (2 × 270 volumes) was recorded using a T2*-weighted gradient echo pulse sequence, echo planar imaging, field of view = 25 cm, matrix size = 96 × 96, slice thickness = 3.4 mm. The volumes covered the whole cerebrum and most of the cerebellum containing 37 slices with 0.5 mm inter-slice gap and an ASSET acceleration factor of 2. The orientation was oblique axial, slices were aligned with the anterior/posterior commissures and scanned in interleaved order with TE = 30 ms, TR = 2 s, flip angle = 80°.

For the Sleep study the first scanning session was following a week with normal sleep, whereas the second session was preceded by a night completely without sleep (see SI methods for further details). The MR imaging was identical to the Dexdor study.

### Behavioural data processing and statistical analyses

In the visuospatial task, trials with response time (RT) < 250 ms or > 2.5 s were excluded prior to statistical analyses. Then, PAS responses between low and high arousal during conscious or suppressed trials were compared using multinomial logistic regression using a Begg-Gray approximation^45^. Afterwards, only trials in absent and suppressed conditions with PAS = 1, and trials with PAS = 3 in the conscious condition, were included in further analyses (SI Results). For the metronome task, the first three trials of each sequence and missed responses were discarded from analysis while all remaining trials were included.

Visual-to-tap asynchrony was calculated as the absolute time difference between stimulus onset and participant’s response. In other words, the smaller the difference, the better the performance. Then, variability in asynchrony was calculated for each sequence and each participant separately for each study.

For the Dexdor Study and the accuracy analyses, a hit was defined as a position match between disc location and the correct response, while an incorrect response was defined as a miss. False alarm (FA) was considered as any responses during the probe presentation for the absent trials. Accuracy was defined as the proportion of (hits-misses). For the Sleep study, a hit was defined as a position match between disc location and probe together with a “yes” response, while a “no” response was defined as a miss. False alarm (FA) was considered as a non-match between disc location and probe with a “yes” response, while a “no” response defined a correct rejection (CR). Accuracy was defined as the proportion of (hits-FA) for conscious and suppressed trials.

Accuracy, under the two arousal levels for both studies, was compared using Wilcoxon’s matched pairs test in conscious and *t- tests* in suppressed conditions. RT differences between the two arousal levels were assessed using repeated measures two-way ANOVA across the three visual presentation conditions. Specific differences for RT in different arousal conditions between suppressed and absent conditions were evaluated using Student’s t-tests.

### fMRI data processing and analyses

For both studies image pre-processing and statistical fMRI data analyses were conducted with SPM12 (Wellcome Department of Imaging Neuroscience, London, UK) running in Matlab 8.4 (Mathworks, Inc., Sherbon, MA, USA) using custom-made Matlab scripts. Functional images were (i) slice-time corrected, (ii) realigned to the first image of the time series to correct for head movement, (iii) unwarped to remove residual movement-related variance, and (iv) co-registered to high-resolution structural data. Structural images were normalized to the MNI (Montreal Neurological Institute) template using DARTEL and resulting parameters were used for functional images normalization, which were resampled to 2-mm isotropic voxel size. Finally, functional images were smoothed with an 8-mm FWHM Gaussian kernel.

Pre-processed data were analyzed using a two-stage summary statistics random effect model. At the first stage, task-dependent changes in BOLD signal were modeled as zero-duration event regressors time-locked to (i) the Mondrians’ onsets for the visuospatial task, including conscious, suppressed, and absent conditions for each arousal level and each PAS rating, and to (ii) the stimulus’ onsets for the visual metronome task, including the four stimulus positions. For the Dexdor study trials where participants fell asleep (no responses for at least three consecutive trials) and the three following trials when participants woke up were discarded from the analysis to avoid biased responses of the sedation with sleep. These regressors were convolved with the SPM12 canonical hemodynamic response function and entered into a general linear model (GLM). The models also included constant terms, six head movement parameters, nuisance regressors such as missed responses, and physiological noise (six parameters) from white matter and cerebrospinal fluid, estimated using the aCompCor method^46^. Finally, high-pass filter (cut-off = 128 s) was applied to remove low-frequency drifts in the data.

Contrast maps were computed on beta maps resulting from the estimated first-level GLMs to reveal for conscious and suppressed conditions, brain regions (i) involved in visuospatial processing and (ii) presenting differences between arousal levels. Individuals’ maps subtending visuospatial networks for conscious and suppressed stimuli were taken to second-level random-effects analyses (one-sample t-tests) to account for inter-individual variability. Comparison between arousal levels was done using repeated-measures ANOVAs for conscious and suppressed conditions. Multiple comparisons correction of statistical maps at the second level was conducted on the whole brain using cluster-based extent thresholding of *p* < 0.05 (FWE corrected) calculated based on the Gaussian random field method and following cluster-defining threshold of *p* < 0.001.

## Supplementary Information

Below is the link to the electronic supplementary material.

Supplementary Material 1

## Data Availability

The datasets generated and analysed during the current study are not publicly available due to ethical restrictions. Access to data by qualified investigators is subject to ethical review and must comply with the European Union General Data Protection Regulation (GDPR) and all relevant guidelines. However, the data are available from the corresponding author upon reasonable request provided a data transfer agreement is signed by an authorized institutional representative.
