# Supplementary material for: Noradrenergic activity as a key target in modulating consciousness
Source: Sci Rep. 2026 Mar 10;16:8729. doi: 10.1038/s41598-026-41819-2 (PMC12979689; doi:10.1038/s41598-026-41819-2)
Supplement: Supplementary file 1 — Supplementary Material 1 [file 41598_2026_41819_MOESM1_ESM.docx]

**SUPPLEMENTARY INFORMATION RESULTS**

**Behavioural results**

| Consc. LS  Consc. MS | % PAS = 1  1.9 (3.0)  5.6 (5.9) | % PAS = 2 % PAS = 3  0.8 (1.5) 97.2 (3.5)  0.8 (1.4) 93.5 (6.3) |
| --- | --- | --- |
| Suppr. LS  Suppr. MS | 79.6 (16.1)  79.7 (13.1) | 19.7 (16.0) 0.7 (1.7)  17.6 (13.2) 1.9 (3.1) |
| Absent LS  Absent MS | 95.2 (7.2)  95.8 (7.2) | 4.3 (6.9) 0.5 (1.0)  3.4 (6.4) 0.6 (1.5) |

RT Conscious RT Suppr. RT Absent

LS 463 (99) 744 (223) 706 (218)

MS 515 (128) 771 (209) 762 (202)

Missing, Consc. Missing, Suppr. Missing, Absent

LS 1.3 (2.6) 1.8 (2.7) 1.3 (2.0)

MS 4.9 (4.8) 6.0 (5.9) 5.3 (5.8)

Table 1. Mean and standard deviation for behavioral variables for the **Dexdor** study. Response time (RT) is reported in milliseconds. “Missing” refers to the average number of trials with no response during the visuospatial task. PAS = Perceptual Awareness Scale; LS = low sedation; MS = moderate sedation; Consc. = conscious trials; Suppr. = suppressed trials.

| Consc. NSD  Consc. SD | % PAS = 1  1.0 (1.8)  6.2 (8.6) | % PAS = 2 % PAS = 3  0.9 (4.7) 98 (5.2)  2.9 (7.7) 91.2 (15.0) |
| --- | --- | --- |
| Suppr. NSD  Suppr. SD | 81.4 (11.1)  83.5(9.1) | 18 (11) 0.6 (0.9)  14.6 (12.9) 1.5 (2.9) |
| Absent NSD  Absent SD | 86.3 (14.4)  90.7 (8.0) | 13.3(14.4) 0.4 (0.9)  8.4(6.9) 0.9 (2.0) |

RT Conscious RT Suppr. RT Absent

NSD 721 (129) 821 (184) 805 (184)

SD 730 (159) 745 (184) 728 (181)

Missing, Consc. Missing, Suppr. Missing, Absent

NSD 0.6 (0.03) 0.9 (0.05) 0.7 (0.03)

SD 4.1 (0.10) 2.6 (0.15) 1.7 (0.08)

Table 2. Mean and standard deviation for behavioral variables for the **Sleep study**. Response time (RT) is reported in milliseconds. “Missing” refers to the average number of trials with no response during the visuospatial task. PAS = Perceptual Awareness Scale; NSD = Non- sleep deprived; SD =Sleep deprived; Consc. = conscious trials; Suppr. = suppressed trials.

For the Dexdor study, during the main visuospatial task, comparison of PAS responses between the sedation levels revealed a significant effect in conscious and suppressed conditions. Specifically, the odds of reporting stimuli as seen (PAS = 3) decreased compared to PAS = 1 during MS (OR = 0.33, *p* = 0.000193) but not compared to PAS = 2 (OR = 1.00, *p* =1.00) for conscious trials. The odds of reporting stimuli as unseen (PAS = 1) did not change compared to PAS=2 (OR = 0.86, *p* = 0.10) nor compared to PAS = 3 (OR = 1.23, *p* = 0.23) whereas the odds of reporting stimuli as seen PAS=3 increased compared to (PAS=2) during MS for suppressed trials (OR = 2.35, *p* = 0.03). The odds of reporting Absent trials as unseen (PAS = 1) did not change between sedation levels (PAS = 1 vs. PAS = 2: OR = 0.75, *p* = 0.20; PAS = 1 vs. PAS = 3: OR = 1.02, *p* = 0.92). For the Sleep study, during the main visuospatial task, comparison between the low and the high arousal levels revealed a significant effect in all conditions. Specifically, the odds of reporting stimuli as seen (PAS = 3) decreased compared to PAS = 1 during low arousal (OR = 0.13, *p* < 0.0001) and compared to PAS = 2 (OR = 0.16, *p* < 0.0001) for conscious trials. The odds of reporting stimuli as unseen (PAS = 1) did not change compared to PAS=2 (OR = 0.89, *p* =0.15) nor compared to PAS = 3 (OR = 1.2, *p* = 0.25) for suppressed trials. The odds of reporting Absent trials as unseen (PAS = 1) decreased compared to PAS 2 (PAS = 1 vs. PAS = 2: OR = 0.67, *p* = 0.000140; but did not change compared to PAS = 3; PAS = 1 vs. PAS = 3: OR = 1.10, *p* = 0.51). See Table 1 and 2 for data. To ensure no conscious visual experience in suppressed trials and clear perception in conscious trials, only trials with PAS = 1 in suppressed and in absent conditions, and trials with PAS = 3 in conscious condition, were included in the analyses.

**SUPPLEMENTARY INFORMATION METHODS**

**Dexmedetomidine sedation: individual adjustment**

The anaesthetic agent used to manipulate the level of arousal was Dexmedetomidine (Dexdor), which activates α_2A_ receptors directly ^1^. To achieve the concentration of 4 µg/ml, 2 ml Dexdor 100 µg/ml was diluted in 48 ml sodium chloride 9 mg/ml. Dexmedetomidine is considered safe and reaches its steady state 15-20 min after infusion ^2^. Due to the pharmacokinetics, we ran the experiment across separate days, with one sedation level for each day. Two sedation levels were used: a moderate level that was adjusted individually, and a low level (0.01 µg/kg/h). The choice of having 0.01 µg/kg/h rather than no sedative or saline injection as a state of comparison was motivated by the fact that the sedative may affect blood flow or other non-neuronal parameters relevant to the fMRI signal ^3,4^. Such effects were also controlled by subtracting BOLD signal related to the absent trials from conscious and suppressed trials.

Individual adjustment of the moderate sedation level was evaluated during a separate pre-scanning session. Participants fasted from solids for at least 6 h and from liquids 4 h before sedation. Dexmedetomidine was infused through an intravenous catheter placed into a forearm vein. Sedation was achieved using computer-controlled intravenous infusion of Dexmedetomidine to obtain constant effect-site concentrations. Participants were initially injected with 0.6 µg/kg/h of Dexmedetomidine. The infusion rate was then increased in steps of 0.1 µg/kg/h, separated by a 20 min stabilization period, until participants were considered moderately sedated, ﻿operationalized as when they showed signs of having difficulties to keep their eyes open (i.e., increased frequency of blinks), but remained responsive in the sense that they could converse with the experimenters and perform the visual metronome task at all times. If participants presented difficulties staying awake (i.e., dozed off, became unresponsive) or had to be prompted to continue performing the metronome task, the sedation level was considered too high, and the previous dose was selected as the appropriate moderate sedation level. Physiological parameters such as blood pressure, heart rate, pulse oximetry, and breathing frequency were continuously monitored and were stable during Dexmedetomidine infusion, and no side effects were observed. Anaesthesia administration and monitoring were based on clinical judgment of the anaesthesiologist and the intensive care nurse. In the final population (n = 25), the range of moderate sedation was 0.6 to 0.8 µg/kg/h (mean ± SD: 0.68 ± 0.07 µg/kg/h).

**Sleep deprivation procedure**

In the morning upon awakening and for seven days in between the first and the second scanning session, participants completed the Consensus Sleep Diary Core version (CSD)^5^. The diary consisted of questions assessing important sleep related information (e.g., bedtime, sleep onset latency, total sleep time, wake time). This helped us to identify and exclude any participants with irregular sleep patterns (poor sleepers) which could lead to biased study results. Participants spent a sleepless night at the university campus where they were staying awake under constant supervision until the next morning. Consumption of alcohol, and intake of caffeine-containing products was prohibited before the second scanning session.


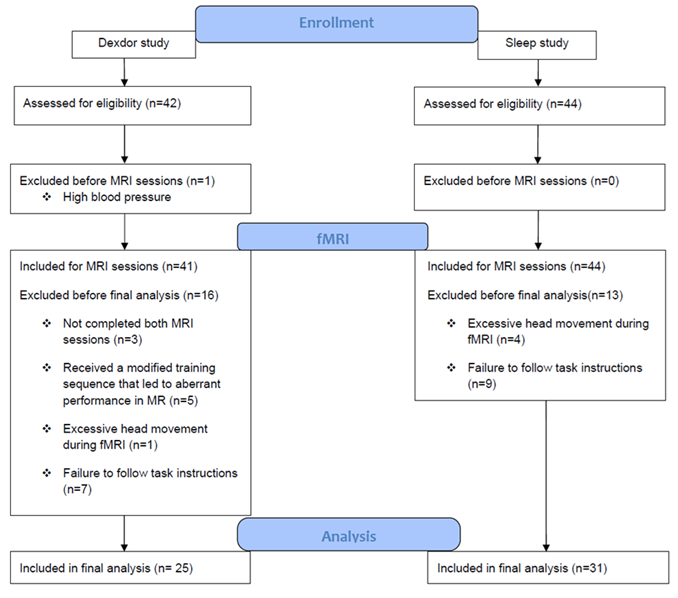


Figure 1. Flow diagram of participants inclusion and exclusion with exact counts per session for Dexdor and Sleep study.

**References**

1. Weerink, M. A. S. *et al.* Clinical Pharmacokinetics and Pharmacodynamics of Dexmedetomidine. *Clinical Pharmacokinetics* **56**, 893–913 (2017).

2. Kaye, A. D. *et al.* Dexmedetomidine in Enhanced Recovery After Surgery (ERAS) Protocols for Postoperative Pain. *Current Pain and Headache Reports* **24**, 21:1–13 (2020).

3. Drummond, J. C. *et al.* Effect of Dexmedetomidine on Cerebral Blood Flow Velocity, Cerebral Metabolic Rate, and Carbon Dioxide Response in Normal Humans. *Anesthesiology* **108**, 225–232 (2008).

4. Fukuda, M., Vazquez, A. L., Zong, X. & Kim, S. Effects of the alfa2-adrenergic receptor agonist dexmedetomidine on neural, vascular and BOLD fMRI responses in the somatosensory cortex. *European Journal of Neuroscience* **37**, 80–95 (2013).

5. Carney, C. E. *et al.* The Consensus Sleep Diary: Standardizing Prospective Sleep Self-Monitoring. *Sleep* **35**, 287–302 (2012).
